# Editorial: Reshaping the diagnostic process in oncology: science versus technology

**DOI:** 10.3389/fonc.2023.1321688

**Published:** 2023-10-24

**Authors:** Fabio Grizzi, Carmen Bax, Laura Capelli, Gianluigi Taverna

**Affiliations:** ^1^ Department of Immunology and Inflammation, IRCCS Humanitas Research Hospital, Rozzano, Italy; ^2^ Department of Biomedical Sciences, Humanitas University, Pieve Emanuele, Italy; ^3^ Politecnico di Milano, Department of Chemistry, Materials and Chemical Engineering “Giulio Natta”, Milano, Italy; ^4^ Department of Urology, Humanitas Mater Domini, Castellanza, Italy

**Keywords:** cancer, diagnosis, science, technology, complexity

The notion that carcinogenesis is a multi-faceted process has now been widely accepted. Nonetheless, only in more recent times have we been able to elucidate a substantial range of molecular circuits that govern the onset and subsequent advancement of human malignancies. Following twenty-five years of accelerated progress, the field of oncology has amassed a complex corpus of data, confirming the idea that cancer is characterized by genomic alterations (1). Despite these significant strides in molecular and cellular comprehension, our grasp of the physical principles that underpin human carcinogenesis remains notably limited (2–4). In recent years, our understanding of the mechanisms behind tumorigenesis, the progression of cancer, and clinical treatments for various types of malignancies has undergone substantial transformations (5). Nevertheless, despite these advancements, considerable challenges still appear large for researchers and oncologists alike. These challenges span a broad spectrum, encompassing the decoding of intricate molecular and cellular processes, the formulation and development of therapeutic strategies and biomarkers, as well as addressing issues related to the quality-of-life following treatment regimens (6). There is no doubt that technological advancements hold a pivotal position in combating cancer. Such innovations have not only transformed the very definition of cancer but have also led to leaps forward in cytological, morphological, and genotypic phenotyping. These developments have consequently given rise to an exponentially expanding array of cancer types and associated care pathways, delineated by their prognostic classifications (7). Indeed, the enhancements in various medical fields, including surgery, radiotherapy, pathology, and imaging, correlated with earlier detection and more effective screening protocols, have served as cornerstones for the betterment of outcomes on a population scale.

According to the National Health System, “diagnosis” is described as the procedure by which a medical condition, disease, or injury is identified through its manifest signs and symptoms. This generally entails accumulating the patient’s medical history, performing a physical assessment, and leveraging a variety of diagnostic tests such as blood analyses, imaging studies, and biopsies to facilitate a conclusive diagnosis. While the notion of “medical diagnosis” can be traced back to eminent historical figures such as the Greek physician Hippocrates (460 BC - 377 BC) and the Egyptian polymath Imhotep (2700 BC - 2630 BC), the existing definition is still marked by certain shortcomings, notwithstanding the plethora of tools and insights offered by contemporary medical research. Medical professionals are increasingly evolving into practitioners who exploit technologies originating from disparate scientific fields, in the expectation that these instruments will alleviate their prevailing uncertainties (8, 9). This period of certainty was constructed upon three fundamental principles: *a)* laws formulated on the basis of deterministic theories coupled with the concept of causality; *b)* the axioms concerning the divisibility and measurability of matter, and *c)* the interlinked notions of stability and reversibility in biological systems. However, this era of certainty in the realm of medicine began to diminish during the 19^th^ century, marked by the emergence of experimental medicine, an approach prominently advocated by the French physiologist Claude Bernard (1813-1878). This development catalyzed intellectual movements that initiated a reevaluation of many overarching conclusions drawn from deterministic theories (10). This critical reassessment ultimately led to the disassembly of various doctrines that were predicated on a strictly mechanistic worldview. Medicine is often described as a practice of decision-making under uncertainty (10).

In the 1950s the philosopher Hans Reichenbach (1891 – 1953) posited that there exists no phenomenon in our Universe that strictly conforms to the principles of *cause* and *effect* (11, 12). The principle of the common cause specifies an important relation between probability and causality. Experimental medicine and biology have also disclosed the presence of biological phenomena and conditions that are observable but defy quantification. The intricate, *non-linear*, and *complex* characteristics inherent to human cancer have propelled numerous researchers to identify innovative biomarkers and to develop advanced histological and imaging methodologies (13).

It is clear that science and technology, while highly interdependent, remain separate yet synergistic domains. Science enriches technology through various channels: *a)* by generating new knowledge that acts as a wellspring for inventive technological prospects; *b)* by offering a repertoire of tools and techniques that streamline the process of engineering design, as well as a foundational knowledge base for assessing the viability of these designs; *c)* by advancing research instrumentation, laboratory methodologies, and analytical techniques that ultimately permeate design or industrial applications, often via intermediary scientific disciplines; *d)* by establishing a comprehensive knowledge repository that fosters more efficacious strategies for the applied research, development, and refinement of emerging technologies. In a reciprocal manner, technology serves science by inspiring new scientific inquiries, thus rationalizing the allocation of resources required to tackle these questions efficiently and promptly.

This Research Topic has garnered contributions in the form of original research and review articles that spotlight avant-garde methodologies to enhance early cancer diagnosis. Hesso et al. have disseminated a segment of the user requirement framework for the INCISIVE EU H2020 initiative, an undertaking architected to fully leverage the capabilities of artificial intelligence (AI)-centric technologies in the realm of cancer imaging. The study was conducted with the aim of elucidating the experiences of cancer survivors with healthcare systems in five European nations. In the current milieu of precision oncology, there has been a growing emphasis on multi-omics data, encompassing imaging radiomics and diverse molecular biomarkers, to refine diagnostic and therapeutic approaches (14). The integration of AI, encompassing machine learning (ML) and deep learning (DL), with the expanding accumulation of multi-omics data, holds considerable promise to bring transformative changes in areas such as cancer subtyping, risk stratification, prognostic evaluations, predictive analytics, and clinical decision-making processes (14). Traditional histopathological methods like immunohistochemistry (IHC)-based receptor tyrosine-protein kinase erbB-2 (Her2) assessment have long been encumbered by intrinsic issues of subjectivity and inconsistency. These challenges have necessitated the recurrent release of guidelines by the American Society of Clinical Oncology/College of American Pathologists (ASCO/CAP) aimed at standardizing the procedure for breast cancer patients. However, these efforts could be rendered inadequate by the introduction of Trastuzumab deruxtecan, a pharmaceutical agent with the potential to be applicable to tumors hitherto classified as Her2-negative. To address these concerns, Yu et al. explored the utilization of an Enzyme-Linked Immunosorbent Assay (ELISA)-akin quantitative dot blot (QDB) methodology as an alternative to IHC. While their results indicate that the QDB method is superior in terms of accuracy and reliability compared to IHC for assessing Her2 protein levels, additional scrutiny is required to assess the viability of the QDB method as an alternative to IHC in addressing the growing demand for identifying tumors with low Her2 expression in routine clinical settings.

In the spectrum of human cancers, glioblastoma multiforme (GBM) is characterized by a high degree of intra-tumoral heterogeneity, evident at both microscopic and radiological levels of resolution (15, 16). Diffusion Weighted Imaging and dynamic contrast-enhanced Magnetic Resonance Imaging are two functional MRI modalities routinely utilized in clinical settings for evaluating the properties of GBM tumors. Brancato et al. provide preliminary findings aimed at ascertaining whether radiomics features (i.e. radiomics, a quantitative approach aiming to extract mineable data from medical images using advanced feature analysis), extracted from preoperative Apparent Diffusion Coefficient (ADC) maps and post-contrast T1-weighted images, correlate with pathomic traits (17) (i.e. pathomics embodies the wide variety of data that is captured from image analyses to generate quantitative features to characterize the describe diverse phenotypic features of tissue samples in high-resolution whole-slide images) discerned from hematoxylin and eosin (H&E) digitized pathology slides. Their findings (Brancato et al.) indicate the potential existence of cross-scale associations between digital pathology metrics and features extracted from ADC and T1c imaging modalities. The implications of these results extend beyond merely enhancing our understanding of the intra-tumoral heterogeneity inherent in GBM. They also serve to bolster the applicability of the radiomics methodology in clinical practice, positioning it as a form of “virtual biopsy.” This could offer fresh perspectives for the integration of omics data in the direction of personalized medical treatments. This somber status is largely attributable to shortcomings in effective early detection mechanisms as well as the constraints of traditional therapeutic options available for patients in advanced stages of the disease (Duan et al.). In recent decades, nanotechnology has risen to prominence as a groundbreaking methodology for achieving desired properties by manipulating objects at the molecular level, garnering significant interest across various medical disciplines. Research indicates that, in the context of lung cancer, nanotechnology-based approaches could offer superior specificity and efficacy in comparison to conventional techniques for the detection of extracellular cancer biomarkers and *in vitro* cancer cells, as well as for *in vivo* cancer imaging.

Duan et al. summarized and analyzed the potential applications of nanotechnology in improving the early diagnosis and precision treatment of lung cancer, intended to provide an adequate theoretical framework for promoting new diagnosis and treatment options. An increasing number of nanomedicines are receiving regulatory approval, showing promising potential for clinical application. Nonetheless, the integration of nanotechnology into clinical practice faces notable challenges and necessitates comprehensive research. Our current knowledge regarding the pharmacokinetics of nanomaterials remains limited. While certain studies indicate that some nanomaterials can maintain their structural integrity *in vivo* for extended periods, the influence of the physical and chemical properties of these nanomaterials on pharmacokinetic bioavailability remains obscure. This lack of understanding complicates the determination of appropriate dosages and administration timing for nanomedicines, potentially leading to unpredictable side effects. Furthermore, despite the significant specificity exhibited by many medical nanomaterials in research, their minute size makes them susceptible to uptake by non-target cells through active transport. This can result in unintended effects or harm to normal tissue cells and may diminish the efficacy of nanomedicine. Despite the rapid progress in nanoscale drug delivery systems, designed to address the obstacles of multi-drug resistance and reduce the impact on healthy tissues by precise delivery to cancerous areas, additional research is required to fully transition nanotechnology-based diagnostic and therapeutic approaches into clinical use.

3D-printed phantoms offer an opportunity to delve deeper into these aspects while also facilitating CT research, particularly by leveraging the capabilities of next-generation scanners. In this Research Topic, Cavaliere et al. introduce a novel anthropomorphic 3D-printed phantom designed for chest lesions. This phantom is customized based on an actual patient’s CT scan and aims to scrutinize the variability in volume and Hounsfield Unit (HU) measurements under diverse CT acquisition conditions. Fused Deposition Modeling (FDM) technology was the chosen printing technique, utilizing polylactic acid (PLA) as the filament material. Comparisons of Dice Similarity Coefficient (DSC) values between the real patient and phantom scans across different kVp settings and on both CT scanners demonstrated substantial overlap in various compartments and in lesion vascularization. Particularly, high similarity was observed for lung and lesion masks in each setting, with DSC values approximately at 0.9 and 0.8, respectively. While the mean HU values could not be directly equated with real patient data due to the use of PLA material, the proportional intensity values for each compartment were maintained. This methodology put forth establishes the reliability of utilizing 3D-printing technology for individualized approaches in CT research, and paves the way for extending this workflow to additional areas in oncology.

Indeed, technology employs the principles of science to address specific challenges, while science utilizes technology to make novel findings (18, 19). Nevertheless, the objectives of science and technology diverge. Science aims to elucidate questions and augment our understanding of phenomena, whereas the purpose of technology is to provide practical solutions to real-world issues. In essence, science is an organized system of acquiring knowledge about the natural universe through systematic methodologies, including data collection. Conversely, technology is the field in which scientific principles are operationalized to develop instruments capable of solving problems and executing various functions. In other words, technology serves as the practical implementation of scientific knowledge, making the two inevitably linked (**Figure 1**). One example of converting scientific knowledge into technological advancement is the exploitation of volatile organic compounds (VOCs) (20–22). Cancer cells release distinct volatile organic compounds (VOCs), which may arise from the oxidative breakdown of polyunsaturated fatty acids due to reactive oxygen species (23). Originally detected by highly trained dogs (24), these compounds can be detected through electronic olfactory devices designed to emulate a dog’s sense of smell (25). This groundbreaking development has promising implications, particularly within the domain of clinical oncology (26). Innovative technological concepts originate from novel scientific discoveries and then proceed through a sequential pathway encompassing applied research, design, production, and ultimately, commercialization and market distribution (27). As technology challenges grow in complexity, the emergence and evolution of technological innovations are not confined to single domains of knowledge but increasingly occur at the intersection of various scientific disciplines and technological domains (28). There exist unresolved questions to this date. Among these, from an ontological standpoint, one must consider the essential nature of both science and technology. How do they parallel or diverge from each other? From an epistemological perspective, questions arise about how knowledge and proficiency are accrued and authenticated in these respective domains. Does technology emerge as a byproduct of scientific exploration, or does science evolve due to technological advancements? Alternatively, could the relationship be reciprocal? Furthermore, is this relationship deterministic in nature, implying that every technological breakthrough necessitates antecedent scientific knowledge? Or is it more probabilistic, suggesting that individuals with a strong scientific background are simply more predisposed to technological innovation?

**Figure 1:**
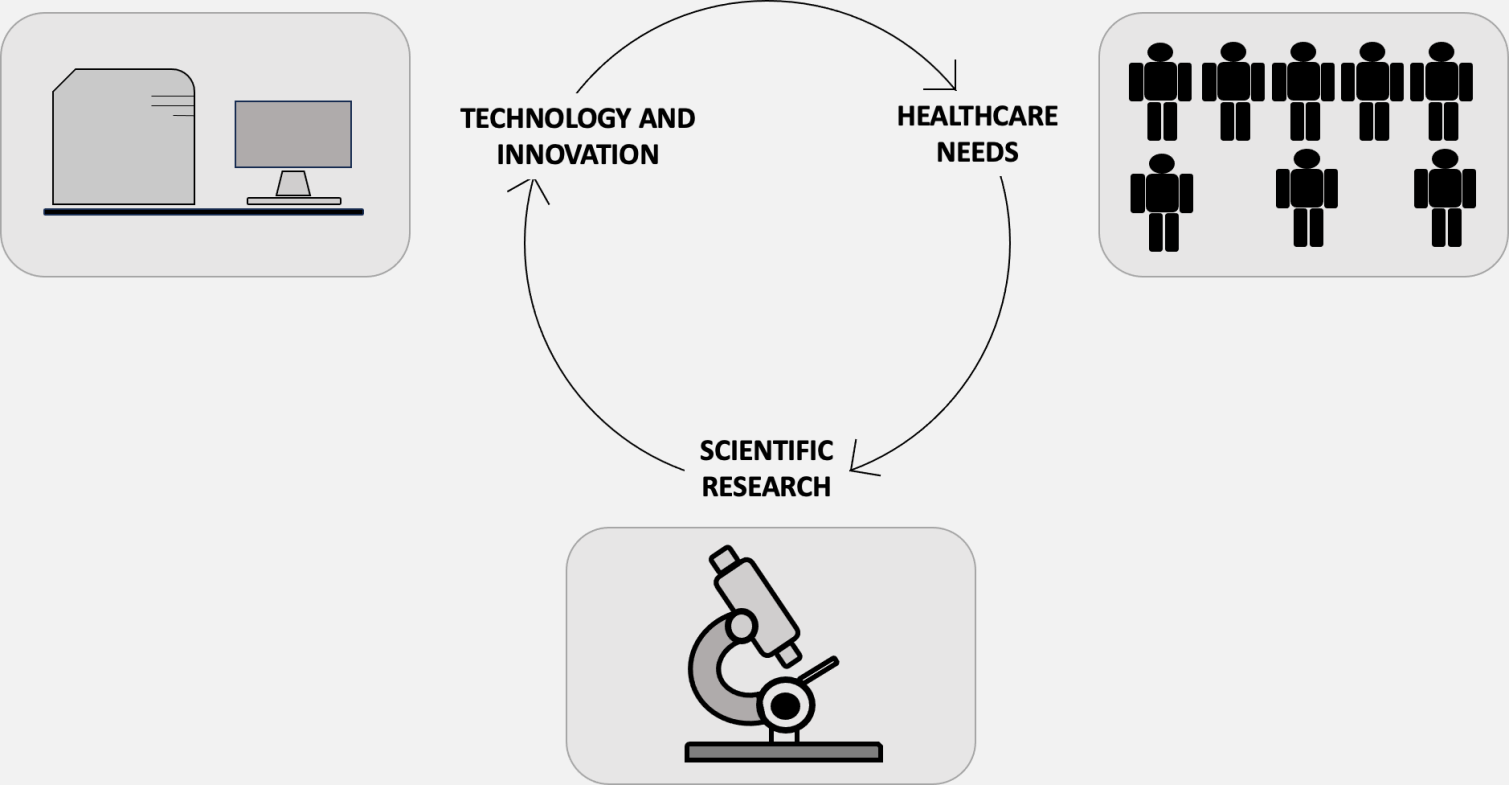
Science, technology, innovation, and health-care demand—each purposed for either maintaining and enhancing health or curing and preventing illnesses—represent expanding realms of activities that are deeply interdependent yet maintain their own unique characteristics. Science serves as a reservoir for engineering design methodologies and techniques. Although the act of design is fundamentally different from the process of uncovering new insights into natural phenomena, the two activities are inextricably intertwined. Conversely, technology emerges as a wellspring for new scientific conundrums, a concept encapsulated by the “chain-link” iterative model, introduced by mechanical engineer Stephen J. Kline in 1985. In this model, the impetus for innovation is not solely the acquisition of new knowledge. Rather, the innovation process is initiated by the identification of an existing gap or need. This identification then propels a sequence of research, design, subsequent redesign, production, and eventually, market deployment.

Certainly, the insights gleaned from the studies included in this Research Topic contribute to an enhanced understanding of the intricate nature of human cancer. Nevertheless, more comprehensive research is imperative for a deeper grasp of the physics underpinning cancer, the modalities of its early detection, and the mechanisms that contribute to therapeutic resistance. Addressing the multidimensional complexity of human cancer, both temporally and spatially, is poised to unveil additional layers of its etiology and progression. This multi-faceted approach may facilitate a more coherent conceptual framework, offer interpretive clarity for experimental data, suggest targeted experiments, and provide a rational means of categorizing the wealth of extant knowledge. It is fundamental that a multi-disciplinary team, including engineers, clinicians, biologists, and mathematicians, continue to collaborate in a concerted effort toward a unified, quantitative comprehension of the complexities of cancer.

## Author contributions

FG: Conceptualization, Supervision, Writing – original draft, Writing – review & editing. CB: Conceptualization, Writing – review & editing. LC: Conceptualization, Writing – review & editing. GT: Conceptualization, Writing – review & editing.
